# The effect of virtual reality-based treadmill gait training on functional mobility and balance in chronic stroke patients: a randomized controlled trial

**DOI:** 10.3389/fneur.2025.1603233

**Published:** 2025-07-21

**Authors:** Seung-Hyeon Han, Hyeon Ju Jang, Jong Weon Lee, Jin Woong Cheong, Young Dae Kim, Hyo Suk Nam, Deog Young Kim

**Affiliations:** ^1^Research Institute of Rehabilitation Medicine, Yonsei University College of Medicine, Seoul, Republic of Korea; ^2^Department of Rehabilitation Medicine, Yonsei University College of Medicine, Seoul, Republic of Korea; ^3^Department of Physical Therapy, Severance Rehabilitation Hospital, Seoul, Republic of Korea; ^4^Department of Neurology, Yonsei University College of Medicine, Seoul, Republic of Korea

**Keywords:** virtual reality, stroke Rehabilitation, gait, postural balance, treadmill training

## Abstract

**Introduction:**

Stroke is a leading cause of long-term disability worldwide. Chronic stroke survivors experience gait and balance impairments, limiting mobility and increasing fall risk. Treadmill training improves walking speed and endurance but lacks real-world adaptability. Virtual reality (VR)-based treadmill training offers immersive, task-specific practice, potentially improving functional mobility in real environments. This randomized controlled trial, using a prospective, open, blinded end-point (PROBE) design, aimed to evaluate the effects of VR-based treadmill gait training on gait performance, functional mobility, balance, and gait symmetry compared with conventional physical therapy in individuals with chronic stroke.

**Methods:**

Thirty chronic stroke patients were randomly assigned to virtual reality treadmill training (VTT) or conventional therapy (CT) groups. Both groups received 30 min of conventional therapy twice weekly for 6 weeks. The VTT group received an additional 30 min of VR-based treadmill training per session using the C-Mill VR+ system, including obstacle negotiation and velocity modulation. The 10-meter walk test (10MWT), 6-min walk test (6MWT), Timed Up and Go (TUG) test, Dynamic Gait Index (DGI), Performance-Oriented Mobility Assessment (POMA), Berg Balance Scale (BBS), asymmetry of spatiotemporal gait parameters, and center of pressure (CoP) sway velocity were assessed pre- and post-intervention.

**Results:**

Walking speed during the 10MWT and the 6MWT significantly increased in the VTT group compared to the CT group, and asymmetric index values for stance and swing duration decreased (*p* < 0.05). TUG test time, DGI values, and the POMA gait component were significantly improved in the VTT group compared to the CT group (*p* < 0.05). BBS scores and CoP sway velocity for eyes open and tandem stance conditions also significantly improved (*p* < 0.05).

**Conclusion:**

This study demonstrated that VR-based treadmill gait training effectively improved gait performance, functional mobility, balance, and gait symmetry in chronic stroke patients. Thus, simulating diverse virtual walking environments in a controlled setting can improve functional benefits in individuals with chronic stroke and potentially reduce fall risk in real-world community and home environments.

**Clinical Trial Registration:**

https://clinicaltrials.gov/study/NCT06557681?term=NCT06557681&rank=1, Identifier: NCT06557681.

## 1 Introduction

Stroke is a leading cause of long-term disability worldwide, with many survivors experiencing persistent gait and balance impairments that hinder independent mobility and daily activities (1). Chronic stroke patients often experience reduced walking speed, asymmetric gait patterns, and impaired balance control, which significantly increase the risk of falls and secondary health complications. These limitations affect physical activity and cardiovascular health, reducing social participation and lowering overall quality of life (2, 3). Thus, various rehabilitation approaches have been introduced to address these challenges. Treadmill-based gait training is widely used to improve walking speed, endurance, and lower limb coordination through repetitive practice (4, 5).

However, conventional treadmill training has inherent limitations, as it primarily focuses on linear, repetitive stepping movements without incorporating the adaptive strategies needed for real-world walking conditions. Effective ambulation requires the constant adjustments of environmental variables, such as obstacle negotiation, directional changes, and maintaining balance during multitasking. Traditional rehabilitation settings often lack the space and resources to simulate these real-world demands in a controlled and safe manner (6, 7).

Virtual reality (VR)-based treadmill gait training has been introduced as an advanced rehabilitation strategy to overcome these challenges. This training creates an interactive, immersive environment where patients can perform task-specific gait exercises with real-time visual feedback. By integrating augmented reality elements, such as obstacle avoidance, variable-speed walking, and balance challenges, VR-based treadmill training can improve motor learning and neuroplasticity through a progressive, adaptable training experience (8). Unlike traditional treadmill training, VR systems may allow patients to practice complex locomotor tasks in a controlled setting, helping them transfer these skills to daily walking activities in real environments (9).

Previous studies have demonstrated the effectiveness of VR-based treadmill training in improving gait function and balance in stroke patients. Heeren et al. (10) reported an open-label clinical trial with a one-group pretest-posttest study design in which VR-based training improved walking speed, balance, and confidence in individuals with chronic stroke. Van Ooijen et al. (11) reported another open-label clinical trial with a one-group pretest-posttest study design where C-mill training improved walking speed, obstacle avoidance and reduced attentional demands during walking. Timmermans et al. (12) reported a randomized controlled case–control study in which C-Mill VR+ training did not improve standard walking speed but improved context-specific walking speed compared to an overground FALLS program, and Yang et al. (13) found that augmented reality treadmill training significantly improved obstacle avoidance and balance, but 10-m walk test did not compared to regular treadmill programs in subacute stroke patients in a randomized, controlled case–control study.

However, the effect of VR-based treadmill training on poststroke patients in the chronic stage remains unclear because randomized control trials (RCTs) focusing on chronic stroke survivors who require targeted interventions to enhance gait adaptability and dynamic balance are lacking (14). Additionally, most prior studies only assessed overall gait and balance scores and obstacle avoidance without conducting detailed analyses of functional mobility, spatiotemporal parameters, and the symmetry of gait or postural stability (e.g., center of pressure velocity and limits of stability) (15).

Thus, this randomized controlled trial, using a prospective, open, blinded end-point (PROBE) design, aimed to comprehensively evaluate the effects of VR-based treadmill gait training using the C-Mill VR+ system on gait performance, functional mobility, and balance in individuals with chronic stroke and compare the clinical efficacy of VR-based treadmill training with conventional physical therapy.

## 2 Methods

### 2.1 Participants

Thirty chronic stroke patients with hemiparesis were recruited for this study from February 2024 to February 2025. All participants were capable of ambulating on level surfaces without physical assistance. The inclusion criteria were: (1) age ≥ 19 years, (2) confirmed diagnosis of stroke using computed tomography or magnetic resonance imaging under the supervision of a neurologist or neurosurgeon, (3) chronic stroke (> 6 months after onset) with hemiparesis, (4) a Functional Ambulatory Category (FAC) score of ≥ 3, and (5) Mini-Mental State Examination (MMSE) score of ≥ 10 points, indicating sufficient cognitive function to comprehend the study protocol, and they voluntarily agreed to participate. The exclusion criteria were: (1) acute or subacute stroke (within 6 months after onset), (2) quadriplegia, (3) ataxia, (4) major co-existing neurological, musculoskeletal, or cardiopulmonary conditions affecting gait, and (5) any other condition deemed unsuitable by the researchers. The participants (mean age 58.7 ± 11.1 years, male-to-female ratio 16:14) had a duration from stroke onset of 85.1 ± 67.7 months, and their average FAC score was 3.43 ± 0.50. All 30 participants completed the study, and no significant differences in baseline characteristic parameters were observed between the two groups. Detailed baseline characteristics, including lesion side, type of stroke, and other clinical information, are presented in Table 1. The study protocol was approved by the Institutional Review Board of the Yonsei University Health System (no. 1-2023-0088). All participants provided written informed consent prior to study enrollment. The study was conducted in accordance with the 2013 revision of the Declaration of Helsinki and was registered at ClinicalTrials.gov (Identifier: NCT06557681).

**Table 1:** Baseline characteristics of subjects.

Variables	All participants (*N* = 30)	CT group (*N* = 15)	VTT group (*N* = 15)	*p*-value^a^
Age (years)	58.7 ± 11.1	60.0 ± 9.9	57.4 ± 12.4	0.532
Sex (M:F)	16:14	6:9	10:5	0.272
Lesion side of stroke (Rt:Lt)	18:12	7:8	11:4	0.264^b^
Type of stroke (Ischemic:Hemorrhagic)	16:14	9:6	7:8	0.715^b^
Duration from onset (month)	85.1 ± 67.7	92.0 ± 64.7	78.3 ± 72.2	0.436^b^
FAC	3.43 ± 0.50	3.47 ± 0.52	3.40 ± 0.51	0.775
MMSE	28.43 ± 1.94	28.67 ± 1.50	28.40 ± 1.72	0.713
10MWT (m/s)	0.34 ± 0.23	0.34 ± 0.29	0.34 ± 0.15	0.325
6MWT (m)	119.78 ± 66.06	114.46 ± 77.23	125.09 ± 54.91	0.345
TUG (s)	28.61 ± 15.30	24.53 ± 12.13	32.70 ± 17.38	0.325
DGI	14.50 ± 2.91	14.67 ± 3.37	14.33 ± 54.91	0.935
POMA total	20.23 ± 1.74	20.47 ± 1.92	20.00 ± 1.56	0.539
Gait part	8.27 ± 0.83	8.40 ± 0.63	8.13 ± 0.99	0.624
Balance part	11.97 ± 1.50	12.07 ± 1.71	11.87 ± 1.30	0.870
BBS	40.73 ± 7.87	41.53 ± 8.48	39.93 ± 7.43	0.412

Values are presented as mean ± standard deviation or number of cases. FAC, Functional Ambulation Category; MMSE, Mini-Mental State Examination; 10MWT, 10-meter walk test; 6MWT, 6-min walk test; TUG, Time Up and Go test; DGI, Dynamic Gait Index; POMA, Performance-Oriented Mobility Assessment; BBS, Berg Balance Scale.

a Statistical analysis was performed using the Mann–Whitney U test to assess group differences.

b Statistical analysis was performed using the Fisher’s exact test to assess group differences.

### 2.2 Samples size calculation

The target sample size was determined *a priori* using the anticipated improvement in the Timed Up and Go test as the primary outcome. Based on post-intervention means and standard deviations reported by Heeren et al. (10) for virtual-reality treadmill training, the pooled-standard-deviation method yielded a Cohen’s d of 0.744. A two-tailed independent-samples t-test in G*Power 3.1 (Heinrich-Heine-Universität Düsseldorf, Germany), with *α* = 0.05 and desired power (1 – *β*) = 0.60, indicated that 14 participants per group (28 in total) were required. Allowing for an anticipated 10% attrition, the target enrolment was increased to 30 participants (15 per group). No dropouts occurred, so the final analyzed sample matched the planned size. The choice of 60% power reflects the exploratory, pilot nature of this randomized controlled trial, which aimed primarily to estimate effect sizes and assess feasibility for a subsequent fully powered study. The planned sample size remained unchanged throughout the trial.

### 2.3 Experimental design

This study employed a PROBE design. The 30 eligible chronic stroke patients were randomly assigned to either the virtual reality treadmill training (VTT) group (*n* = 15) or the conventional therapy (CT) group (*n* = 15) using a random number table generated in SPSS (version 27.0, IBM Corp., Armonk, NY, United States). The study followed a single-blind methodology, where the therapists administering the assessment were blinded to the participants’ group allocations.

After randomization, all participants underwent a baseline assessment prior to the intervention. Both groups received training sessions twice per week for 6 weeks for a total of 12 sessions. The CT group received 30 min of conventional rehabilitation therapy focused on gait and balance. In contrast, the VTT group received the same 30-min conventional therapy, then an additional 30-min VR-based treadmill training session. In the VTT group, the sequence of conventional therapy and VR treadmill training was allowed to vary across sessions, as the order was not considered to affect the therapeutic outcomes.

The VTT group participated in a structured, therapist-supervised program consisting of five key components designed to enhance gait adaptability and balance: (A) precision stepping, (B) obstacle negotiation, (C) direction of progression, (D) precision acceleration, and (E) walking velocity modulation.

In contrast, the CT group underwent therapist-directed conventional rehabilitation, which included interventions tailored to improve muscle strength, gait parameters (step length and walking speed), and balance control. The treating therapist determined the specific components of the conventional therapy based on individual patient needs.

Outcome measures of gait and balance were assessed before and after the interventions, with the post-assessment performed within 3 days of training program completion. The same trained evaluator, who was not involved in providing the intervention, conducted all assessments to ensure consistency. Different therapists administered the interventions and performed the assessments, ensuring the objectivity of the evaluation process. Both groups completed all interventions without any adverse events.

### 2.4 Virtual reality treadmill-based gait training

**Figure 1:**
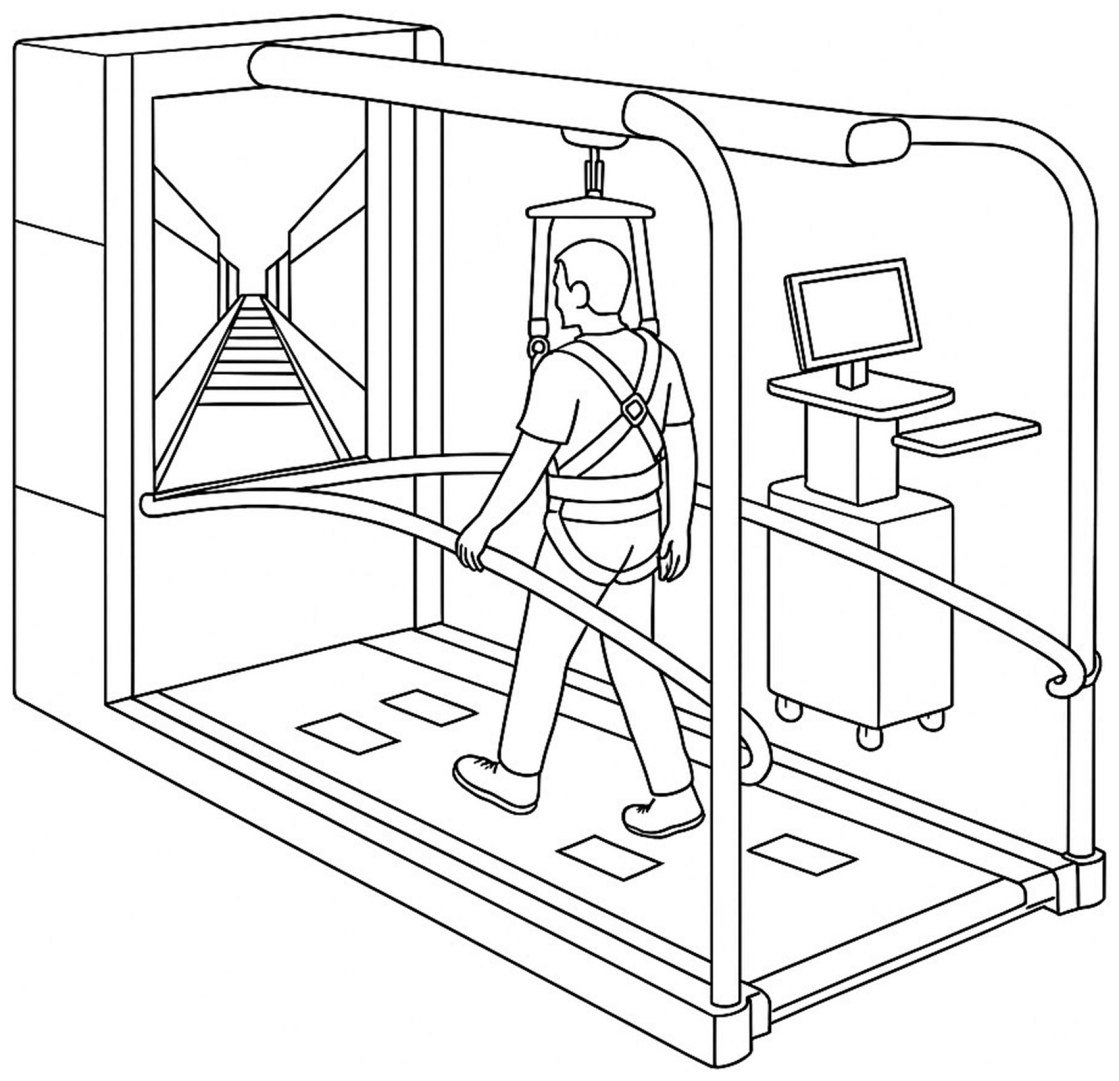
C-Mill VR+ instrumented treadmill used for the virtual-reality gait training protocol.

**Figure 2:**
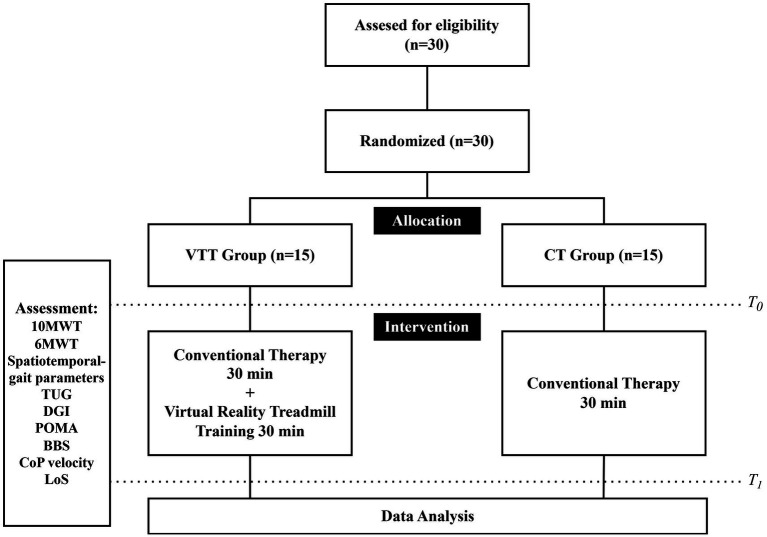
Study process flow.

C-Mill VR+ (Motek Medical, Amsterdam, Netherlands) was utilized for both the assessments and interventions in this study (10). C-Mill VR+ is a VR-based treadmill system that integrates a front-facing screen and visual projections on a treadmill belt, providing automated, standardized, and patient-tailored gait adaptability training (12, 16). All subjects received a thorough explanation of the system before participating in the assessment and training program and underwent an adaptation period to ensure familiarity with the C-Mill VR+ environment. All training were supervised by experienced therapists, and the participants wore a safety harness mounted on the device to prevent accidental falls. The walking speed was determined before each training session by gradually increasing the treadmill speed in 0.1 km/h increments until the participant reported their self-selected comfortable walking speed. The treadmill dynamically monitored gait parameters in real-time, adjusting the VR-based targets and obstacles presented on the treadmill surface to provide task-specific gait challenges. The participants were instructed not to use the handrails during the assessment and intervention sessions, except in cases where fall prevention was necessary for safety. The training program’s difficulty was designed to progressively adjust according to the participant’s functional improvement, ensuring a gradual and adaptive training intensity (see Figures 1, 2).

### 2.5 Outcome measures

To evaluate the effects of VR-based treadmill training, gait performance, functional mobility and balance were assessed before and after the intervention. Gait performance was assessed using the 10-meter walk test (10MWT), the 6-min walk test (6MWT), spatiotemporal gait parameters, and the Asymmetry Index (AI). Functional mobility was assessed using the Timed Up and Go (TUG) test, Dynamic Gait Index (DGI), and the Performance-Oriented Mobility Assessment (POMA). Balance was assessed using the Berg Balance Scale (BBS), center of pressure (CoP) sway velocity, and limits of stability (LoS).

In the 10MWT, tape was placed at the 2- and 8-meter marks of a 10-meter walkway. The participants completed three trials at their self-selected comfortable speed. The time to traverse the central 6 meters was recorded using a stopwatch, and the fastest time was used for analysis. The test provides a reliable measure of walking speed, which is a key indicator of overall gait performance.

The 6MWT was conducted to measure gait endurance. It was performed on a straight 12-meter course marked by cones at each end. The participants were instructed to walk back and forth continuously for 6 min. The total walking distance, including turns, was measured using a measuring wheel. The assessor followed at a safe distance to ensure precise measurement and participant safety.

Spatiotemporal gait parameters were collected using the C-Mill VR+ treadmill system, which continuously monitored step length, step time, and stance-swing phase distribution during the training sessions. Gait symmetry was analyzed using AI values. Step length and stance duration were compared between the affected and unaffected limbs. AI values were calculated using the following equation: AI in % = |[(unaffected side-affected side)/0.5*(unaffected side+affected side)]| * 100 (17). A lower AI value indicates a reduction in differences between the affected and unaffected limbs and, thus, improved gait symmetry. These parameters were used to evaluate gait performance and efficiency.

In the TUG test, the participants were instructed to rise from a seated position, walk 3 meters, turn around, return to the chair, and sit down as quickly and safely as possible. The time to complete the task was recorded, with shorter times indicating better functional mobility and balance control. The test was performed three times, and the fastest recorded time was used for analysis. The TUG test is widely utilized to assess fall risk and functional ambulation, with longer completion times associated with higher fall risk and decreased dynamic balance in stroke survivors (18).

The DGI was used to assess functional mobility and dynamic balance during walking by evaluating the performance of tasks, such as walking with speed changes, pivot turns, and obstacle negotiation. The index consists of eight tasks, including walking while turning the head in different directions and stepping over obstacles. The total scores range from 0 to 24, with higher scores indicating better dynamic gait stability and a lower risk of falls in stroke survivors (19).

The POMA was used to evaluate the capacity to perform functional mobility and balance. The assessment consists of a 16-item assessment that evaluates gait (12 points) and balance (16 points), with a maximum score of 28. It examines components such as step symmetry, step continuity, standing balance, and response to external perturbations. The POMA provides a comprehensive evaluation of both static and dynamic mobility, making it a valuable tool for assessing fall risk and overall functional movement. Higher scores indicate better postural control and gait stability, with a lower likelihood of fall-related impairments (20).

The BBS was used to assess both static and dynamic balance control. The participants were required to complete 14 balance-related tasks, including standing on one leg, turning 360 degrees, and reaching forward while maintaining stability. Each task was rated on a scale of 0 to 4, with a higher total score indicating better balance control. The BBS is widely used in clinical settings to predict fall risk and evaluate the effectiveness of balance training interventions.

In this study, postural stability and LoS were measured using the force plate built into the C-Mill VR+ treadmill (21). Postural stability was evaluated under four conditions (eyes open, eyes closed, tandem stance, and single-leg stance) by analyzing velocity (cm/s) and CoP area (cm^2^), where lower values indicate better static balance (22). For the LoS assessment, the participants were instructed to maximally shift their weight in mediolateral (left–right) and anterior–posterior (forward-backward) directions. The maximum CoP displacement was recorded to reflect dynamic balance capacity (23–25). Each direction was tested four times, and the mean values were used for analysis. Larger LoS values indicate a broader safe range for weight shifting, suggesting improved dynamic balance and potentially reduced fall risk (22).

A minimum of 5 min of rest was provided between assessments to minimize fatigue. All evaluations were conducted under the supervision of an experienced therapist to ensure participant safety.

### 2.6 Statistical analysis

All statistical analyses were conducted using SPSS software (version 27.0, IBM Corp., Armonk, NY, USA). Data distribution normality was assessed using the Shapiro–Wilk test. Descriptive statistics were generated for all outcome measures and presented as means and standard deviations (mean ± SD). Within-group comparisons were performed by analyzing pre- and post-intervention differences (*Δ* values). Paired t-tests were used for normally distributed data, while Wilcoxon signed-rank tests were applied for non-normally distributed data. A generalized linear mixed model (GLMM) was employed to evaluate the effects of group (VTT vs. CT) and time (pre- vs. post-intervention), incorporating random effects for individual participants to account for inter-subject variability. The GLMM was selected as it allows for the analysis of repeated measures data, including both fixed effects (group and time) and random effects (individual differences). The model was used to assess interaction effects (group × time) to determine the differential impacts of the interventions between groups. This approach provides a more robust estimate of the intervention’s efficacy than traditional repeated measures analysis of variance (ANOVA) by accommodating missing data and adjusting for within-subject correlations. A significance threshold of *p* < 0.05 was applied for all statistical tests. Bonferroni correction was applied to control for type I errors in pairwise post-hoc analyses. Cohen’s *d* was calculated for all continuous outcomes. The statistic equals the between-group difference in mean change scores divided by the pooled baseline standard deviation. A positive *d* indicates that the VTT group improved more than the CT group on outcomes where higher scores reflect better performance, whereas a negative *d* indicates greater improvement in the VTT group on outcomes where lower scores reflect better performance. Effect sizes were classified as small when |*d*| < 0.5, medium when 0.5 ≤ |*d*| < 0.8, and large when |*d*| ≥ 0.8.

## 3 Results

### 3.1 Gait performance

Walking speed in the 10MWT significantly improved in both groups after the intervention; however, the VTT group showed a significantly greater increase than the CT group (*Δ* 0.07 vs. 0.04 m/s, *p* = 0.003). The 6MWT distance significantly increased post-intervention in both groups, with the VTT group demonstrating a significantly greater improvement than the CT group (*Δ* 19.79 vs. 10.19 m, *p* = 0.005) (Table 2).

**Table 2:** Comparison of gait performance after intervention between CT and VTT groups (*n* = 30).

	CT group (*n* = 15)			VTT group (*n* = 15)				
Parameter	Pre-intervention	Post-intervention	Δ	Pre-intervention	Post-intervention	Δ	*d*	*p*-value^a^
10MWT (m/s)	0.33 ± 0.27	0.37 ± 0.29*	0.04	0.32 ± 0.14	0.39 ± 0.15*	0.07	0.14	0.003
6MWT (m)	115.5 ± 72.83	125.69 ± 69.92*	10.19	119.01 ± 52.43	138.8 ± 54.21*	19.79	0.15	0.005

Values are presented as mean ± standard deviation. CT, conventional therapy; VTT, virtual reality-treadmill training. *Statistically significant within-group (Wilcoxon signed-rank test, *p* < 0.05).

a Statistical analysis was performed using a generalized linear mixed model to examine group-by-time interaction.

### 3.2 Spatial–temporal walking parameters

The step length on the affected side significantly increased in the VTT group compared to the CT group (*Δ* 0.07 vs. 0.01 m, *p* = 0.049). No significant differences between groups were observed in stance duration, swing duration, single support time duration, double support duration, or step length on the unaffected side (*p* > 0.05) (Table 3).

**Table 3:** Comparison of Spatial–temporal walking parameters after intervention between CT and VTT groups (*n* = 30).

		CT group (*n* = 15)			VTT group (*n* = 15)				
Parameter		Pre-intervention	Post-intervention	Δ	Pre-intervention	Post-intervention	Δ	*d*	*p*-value^a^
Affected side	Step length (m)	0.27 ± 0.03	0.28 ± 0.02	0.01	0.27 ± 0.08	0.33 ± 0.07*	0.07	0.99	0.049
	Stance duration (%)	68.60 ± 1.47	68.87 ± 1.54	0.27	68.93 ± 2.57	69.53 ± 1.93	0.60	0.16	0.899
	Swing duration (%)	31.40 ± 1.47	31.13 ± 1.54	−0.27	31.07 ± 2.57	30.47 ± 1.93	−0.60	−0.16	0.899
	Single support stance (%)	26.60 ± 1.32	27.47 ± 1.41	0.87	23.33 ± 2.01	26.40 ± 1.38	3.07	1.29	0.224
Unaffected side	Step length (m)	0.30 ± 0.02	0.31 ± 0.02	0.01	0.26 ± 0.02	0.29 ± 0.02	0.04	1.50	0.339
	Stance duration (%)	73.40 ± 1.32	72.53 ± 1.41	−0.87	76.67 ± 2.01	73.60 ± 1.38	−3.07	−1.29	0.224
	Swing duration (%)	26.60 ± 1.32	27.47 ± 1.41	0.87	23.33 ± 2.01	26.40 ± 1.38	3.07	1.29	0.224
	Single support stance (%)	31.40 ± 1.47	31.13 ± 1.54	−0.27	31.07 ± 2.57	30.47 ± 1.93	0.60	−0.16	0.899
	Double support duration (%)	43.20 ± 1.77	41.67 ± 2.16	−1.53	45.20 ± 2.7	44.40 ± 2.87	0.80	0.32	0.758
	Stance duration difference (%)	4.80 ± 8.47	3.67 ± 7.95	−1.13	7.73 ± 14.78	4.07 ± 7.05	3.67	−0.21	0.525
	Step length difference (m)	0.04 ± 0.14	0.03 ± 0.11	−0.01	0.01 ± 0.12	0.04 ± 0.07	0.03	0.31	0.377

Values are presented as mean ± standard deviation. CT, conventional therapy; VTT, virtual reality-treadmill training. *Statistically significant within-group (paired t-test, *p* < 0.01).

a Statistical analysis was performed using a generalized linear mixed model to examine group-by-time interaction.

### 3.3 Asymmetry index values for gait parameters

The AI values for stance duration in the VTT group significantly improved compared to the CT group (*Δ* −8.6 vs. −1.8, *p* = 0.049). The AI values for swing duration also significantly improved in the VTT group compared to the CT group (*Δ* −24.3 vs. −5.5, *p* = 0.036). The stance AI values for single support showed a significant improvement in the VTT group compared to the CT group (*Δ* −24.3 vs. −5.5, *p* = 0.036). No significant differences were seen between groups in AI values for step length (*p* = 0.688) (Table 4).

**Table 4:** Comparison of asymmetry index for gait parameters after intervention between CT and VTT groups (*n* = 30).

	CT group (*n* = 15)			VTT group (*n* = 15)				
Parameter	Pre-intervention	Post-intervention	Δ	Pre-intervention	Post-intervention	Δ	*d*	*p*-value^a^
Step length (%)	43.2 ± 29.2	33.2 ± 23.2	−10.0	38.3 ± 27.2	24.3 ± 23.0*	−14.0	0.14	0.688
Stance duration (%)	10.2 ± 8.6	8.4 ± 9.1	−1.8	18.1 ± 14.1	9.4 ± 6.0*	−8.6	0.58	0.049
Swing duration (%)	26.3 ± 22.8	20.8 ± 21.3	−5.5	48.8 ± 35.1	24.5 ± 16.1*	−24.3	0.64	0.036
Single support time duration (%)	26.3 ± 22.8	20.8 ± 21.3	−5.5	48.8 ± 35.1	24.5 ± 16.1*	−24.3	0.64	0.036

Values are presented as mean ± standard deviation. CT, conventional therapy; VTT, virtual reality-treadmill training. *Statistically significant within-group (Wilcoxon signed-rank test, *p* < 0.05).

a Statistical analysis was performed using a generalized linear mixed model to examine group-by-time interaction.

### 3.4 Functional mobility

The TUG test time significantly decreased in the VTT group compared to the CT group after the intervention (*Δ* −6.67 vs. −2.18 s, *p* = 0.012). DGI scores improved significantly in both groups, with the VTT group exhibiting a significantly greater increase than the CT group (Δ 4.22 vs. 1.94, *p* = 0.000). Scores on the POMA gait components were significantly improved in both groups, with the VTT group demonstrating a significantly greater increase compared to the CT group (*Δ* 0.94 vs. 0.50, *p* = 0.014). However, the total POMA and POMA balance scores were not significantly different between the groups (*p* = 0.172 and *p* = 0.725, respectively) (Table 5).

**Table 5:** Comparison of functional mobility after intervention between CT and VTT groups (*n* = 30).

	CT group (*n* = 15)			VTT group (*n* = 15)				
Parameter	Pre-intervention	Post-intervention	Δ	Pre-intervention	Post-intervention	Δ	*d*	*p*-value^a^
TUG (s)	25.02 ± 11.49	22.84 ± 10.60*	−2.18	30.33 ± 16.83	23.65 ± 11.85*	−6.67	−0.31	0.012
DGI	14.56 ± 3.13	16.5 ± 3.33*	1.94	14.39 ± 2.3	18.61 ± 1.82*	4.22	0.83	0.000
POMA total	20.17 ± 1.95	21.61 ± 1.69*	1.44	20.17 ± 1.5	22.22 ± 1.83*	2.06	0.36	0.172
Gait part	8.28 ± 0.83	8.78 ± 0.55*	0.50	8.22 ± 0.94	9.17 ± 0.51*	0.94	0.50	0.014
Balance part	11.89 ± 1.60	12.83 ± 1.47*	0.94	11.94 ± 1.3	13.06 ± 1.63*	1.11	0.12	0.725

Values are presented as mean ± standard deviation. CT, conventional therapy; VTT, virtual reality-treadmill training. *Statistically significant within-group (Wilcoxon signed-rank test, *p* < 0.05).

a Statistical analysis was performed using a generalized linear mixed model to examine group-by-time interaction.

### 3.5 Balance

BBS scores improved significantly in both groups, with significantly greater increases in the VTT group (*Δ* 4.33 vs. 2.44, *p* = 0.005). The eyes-open sway velocity significantly decreased in the VTT group compared to the CT group (Δ −0.95 vs. 0.15 cm/s, *p* = 0.029). The tandem stance sway velocity also significantly decreased in the VTT group compared to the CT group (Δ −2.46 vs. −0.02 cm/s, *p* = 0.030). No significant differences were found between groups in eyes-closed sway velocity, one-leg stance sway velocity, CoP area, or mediolateral/anterior–posterior range (*p* > 0.05) (Table 6).

**Table 6:** Comparison of postural stability after intervention between CT and VTT groups (*n* = 30).

	CT group (*n* = 15)			VTT group (*n* = 15)				
Parameter	Pre-intervention	Post-intervention	Δ	Pre-intervention	Post-intervention	Δ	*d*	*p*-value^a^
BBS	41.17 ± 7.76	43.61 ± 7.72*	2.44	39.39 ± 9.02	43.72 ± 8.33*	4.33	0.23	0.005
CoP sway velocity								
Eyes open (cm/s)	5.28 ± 2.11	5.42 ± 2.02	0.15	6.09 ± 2.23	5.14 ± 1.78*	−0.95	−0.51	0.029
Eyes close (cm/s)	6.64 ± 3.45	5.89 ± 2.45	−0.75	6.51 ± 2.47	5.31 ± 1.61*	−1.19	−0.15	0.477
Tandem stance (cm/s)	8.55 ± 3.24	8.53 ± 3.73	−0.02	10.09 ± 4.39	7.64 ± 2.54*	−2.46	−0.63	0.030
One leg stance (cm/s)	18.75 ± 13.35	15.24 ± 7.22	−3.50	20.29 ± 11.13	13.91 ± 7.62*	−6.38	−0.23	0.443
Limits of stability								
CoP area (cm^2^)	91.25 ± 31.26	114.52 ± 23.66*	23.26	93.83 ± 44.96	126.21 ± 49.04*	32.38	0.24	0.464
ML range of CoP (cm)	15.83 ± 4.03	18.91 ± 3.18*	3.08	16.23 ± 5.08	18.78 ± 4.87*	2.55	−0.12	0.741
AP range of CoP (cm)	11.02 ± 2.10	12.13 ± 1.60*	1.12	10.82 ± 3.11	12.60 ± 2.61*	1.78	0.25	0.378

Values are presented as mean ± standard deviation. CT, conventional therapy; VTT, virtual reality-treadmill training; CoP, center of pressure; ML, mediolateral; AP, anterior–posterior. * Statistically significant within-group (Wilcoxon signed-rank test, *p* < 0.05).

a Statistical analysis was performed using a generalized linear mixed model to examine group-by-time interaction.

## 4 Discussion

This study aimed to comprehensively evaluate the effects of VTT on gait performance, functional mobility, and balance in chronic stroke survivors. The findings demonstrated significant improvements in gait performance, as reflected by increased walking speed (10MWT), enhanced walking endurance (6MWT), improved spatiotemporal gait parameters, and reduced gait asymmetry. Additionally, the VTT participants exhibited greater improvements in functional mobility (TUG, DGI, and POMA gait subscale), as well as enhanced balance (BBS and CoP sway velocity), compared to those receiving conventional therapy alone. These results support the potential of VTT as an effective rehabilitation intervention for improving functional gait and balance control in chronic stroke survivors.

This study results demonstrated significant improvements in gait performance following VTT, including increased walking speed (10MWT) and enhanced walking endurance (6MWT). It is consistent with previous RCTs that reported increased walking speed following VR-based treadmill training in stroke patients (13, 26, 27). Specifically, Wang et al. (26) demonstrated superior improvements in walking speed after VR treadmill training compared to conventional therapy. De Melo et al. (28) showed greater enhancements in walking endurance, assessed by the 6MWT in Parkinson’s disease patients who underwent VR treadmill training compared to those who received conventional therapy. These results highlight the beneficial effects of VR-based training on key gait performance parameters, suggesting improved functional walking capacity and endurance for daily activities and community ambulation.

This study also demonstrated significant improvements in gait symmetry in the VTT group, as evidenced by significant reductions in asymmetry indices for stance duration, swing duration, and single support time duration compared to the CT group. Previous studies have consistently reported improved gait symmetry following visual feedback treadmill training. Drużbicki et al. (29) reported that chronic stroke patients showed sustained improvements in stance and swing duration symmetry following visual biofeedback treadmill training. Similarly, Jo et al. (30) demonstrated significant reductions in step-length asymmetry in chronic stroke survivors after visual feedback training emphasizing anterior–posterior weight shifting. Although traditional gait training approaches may partially improve gait symmetry, they generally lack individualized, precise feedback mechanisms. Hesse et al. (31) reported limited and non-significant improvements in gait symmetry indices in stroke patients following conventional neurodevelopmental therapy. The limited effectiveness of conventional approaches may result from repetitive practice without personalized or precise visual and kinematic feedback. These findings support our hypothesis that real-time visual and kinematic feedback, as well as individualized and interactive gait tasks provided within the VR environment, actively promoted more symmetrical gait patterns by encouraging patients to utilize their affected limbs more effectively. Improvements in gait symmetry are clinically meaningful because symmetrical gait patterns are strongly associated with increased walking efficiency and reduced energy expenditure. Awad et al. (32) showed that decreasing step-length asymmetry significantly reduced the energy cost of walking in stroke patients, resulting in more efficient gait patterns. Wert et al. (33) further supported these findings by demonstrating that reduced energy expenditure during walking correlated with greater functional independence. Therefore, the significant improvements in gait symmetry observed in our study suggest that VTT may not only enhance the quality of gait but also contribute to long-term improvements in overall functional independence and mobility in chronic stroke patients.

Functional mobility, assessed through the TUG test, the DGI, and the POMA gait component, significantly improved after VTT in this study. Improvements in the TUG scores are particularly meaningful, as this measure closely reflects daily ambulatory activities involving transitional movements such as standing up, walking, turning, and sitting down. Previous studies consistently reported improvements in TUG performance following VR treadmill or gait training in stroke patients (10, 12, 26), Parkinson’s disease (34), and older adults (35). TUG performance is also closely associated with fall risk, with shorter completion times reflecting a reduced likelihood of future falls among older adults living in community settings (36). The significant TUG improvements observed in our study suggest that VTT effectively enhanced the participants’ abilities to perform complex, real-world functional movements safely and efficiently. Additionally, substantial improvements observed in the DGI scores and the gait component of the POMA in the VTT group indicate enhanced dynamic balance and gait adaptability. Timmermans et al. (12) previously reported significant improvements in context-specific obstacle negotiation using the C-Mill VR environment in stroke patients. Wang et al. (26) reported superior improvements in obstacle avoidance success rates on treadmill walking from VR-based training compared to conventional training. Similar improvements were observed in our study, highlighting the advantage of the VR training environment, which allowed patients to repeatedly practice realistic gait scenarios involving obstacle avoidance, variable walking speeds, and directional changes. Such training conditions likely facilitated improved gait adaptability, enabling the participants to transfer learned gait strategies effectively to real-life walking environments. These outcomes reinforce the importance of context-specific repetitive training in enhancing functional mobility, dynamic balance, and overall walking adaptability among chronic stroke survivors.

This study also demonstrated significant improvements in balance and postural stability following VTT. The participants in the VTT group showed greater improvements in functional balance measured by the BBS than those in the CT group. Additionally, significant reductions in sway velocity during challenging postural conditions, such as tandem stance, indicated enhanced dynamic balance control. These findings align closely with previous studies reporting similar improvements in balance and postural control in chronic stroke survivors who underwent VR treadmill training compared to conventional treadmill interventions (10, 37). Yang et al. (37) and Heeren et al. (10) reported greater improvements in dynamic balance and postural control in stroke patients assessed by the BBS following VR treadmill training compared to conventional treadmill training. Shin and Chung (38) demonstrated significant gains in balance functions when visual and auditory feedback were integrated into treadmill training. Collectively, these results suggest that the real-time visual and kinematic feedback provided by the VR environment facilitated more precise weight shifting and postural control strategies, contributing to improved balance performance observed in this study.

Notably, the significant reduction in CoP sway velocity during eyes-open conditions indicates that VTT specifically improved the patients’ abilities to effectively utilize visual feedback for postural stabilization. These findings are supported by Zhang et al. (39), who reported that visual feedback-based training improved weight distribution control and overall postural stability. Reduced CoP sway velocity observed in the tandem stance condition suggests enhanced core muscle stability and coordination ability from VR-based treadmill training. The task-specific challenges incorporated in the VTT, including obstacle negotiation and directional changes, likely promoted core muscle activation and improved overall trunk stability, both of which are critical for the successful execution of complex real-world gait tasks. Consistent with these findings, El-Nashar et al. (40) found that core-strengthening exercises significantly improved dynamic sitting balance and trunk stability in chronic stroke patients. Thus, the current findings highlight the benefit of VR treadmill training in enhancing trunk control and dynamic balance required during practical activities, such as turning and navigating obstacles. However, improvements in the LoS and the balance component of POMA under static balance conditions were not significant in this study. This limitation may be due to the VTT protocol, which primarily focused on dynamic gait tasks and visual information processing rather than static balance or sensory integration without visual cues. Hwang et al. (41) previously demonstrated that visual feedback training was effective in improving both static and dynamic balance in stroke patients by reducing CoP sway velocity and increasing LoS. Therefore, future VR-based training interventions should consider integrating proprioceptive and vestibular sensory inputs to further enhance comprehensive balance outcomes.

Although the post-intervention DGI approached the conventional fall-risk threshold, participants’ scores remained sufficiently below the scale maximum to rule out a true ceiling effect. The gain in DGI coincided with a reduction in CoP sway when visual input was available, while eyes-closed and LoS conditions showed no change. These findings suggest that the current VR protocol strengthened visually guided, feed-forward control of gait and posture rather than recalibrating proprioceptive or vestibular inputs. To promote broader multisensory integration and improve real-world transfer, future VR programs should incorporate tasks that challenge these additional sensory systems, such as reduced-vision walking, compliant or perturbing surfaces, and head-movement exercises.

Clinically, chronic stroke rehabilitation is often challenging due to the perceived plateau in recovery, whereby functional improvements tend to decrease over time (42). Nonetheless, this study provides strong evidence supporting that intensive, task-specific gait training through VR technology can promote neuroplasticity and facilitate motor relearning even in the chronic stage following stroke. Previous research by Teasell et al. (43) and Page et al. (44) indicated that targeted, intensive rehabilitation interventions could significantly enhance functional recovery in chronic stroke patients, regardless of time since stroke onset. Numerous other studies have consistently demonstrated the potential for meaningful improvements in long-term chronic stroke survivors (45, 46). The immersive and interactive characteristics of VR-based treadmill training enable the progressive, adaptive practice of various gait tasks, which may explain the significant gains in gait adaptability, symmetry, and postural control observed despite chronicity in this study. Furthermore, while prior studies largely focused on basic measures such as walking speed and endurance, the present study incorporated more comprehensive gait symmetry and postural stability assessments, thereby providing a broader understanding of gait recovery and functional restoration in chronic stroke patients.

In typical clinical settings, conventional gait rehabilitation often requires large spaces and carefully structured environments for the safe practice of real-world ambulation tasks, posing practical challenges for clinicians. VR-based treadmill training effectively overcomes these limitations by providing a controlled yet highly varied gait training environment within a limited physical space. Patients can repeatedly practice realistic walking scenarios, such as obstacle avoidance, speed variation, and directional changes, which are essential for transferring these acquired skills to real-world mobility. This is particularly beneficial for chronic stroke survivors who may encounter difficulty accessing community-based gait training due to environmental restrictions or fear of falling (47, 48). The study findings suggest that VR-based treadmill training can be a valuable rehabilitation tool for long-term stroke survivors with lower ambulatory function, providing a safe, engaging, and task-specific intervention.

From a physiological standpoint, four mutually reinforcing mechanisms may account for the superior gait gains produced by VR-based treadmill training. First, the coordinated delivery of optic flow, proprioceptive loading, and vestibular input strengthens multisensory integration within fronto-parietal–cerebellar networks, sharpening step-to-step error detection and correction (49). Second, the immersive, task-specific scenarios heighten attentional engagement and practice intensity; functional-MRI studies have consequently demonstrated up-regulation of the ipsilesional sensorimotor cortex and supplementary motor area after VR-enhanced gait practice (50). Third, the moderate-to-vigorous walking bouts intrinsic to VR treadmill sessions acutely elevate circulating brain-derived neurotrophic factor, a neurotrophin that supports synaptic remodeling and long-term motor recovery (51). Finally, repeated exposure to variable step lengths, obstacle negotiation, and speed shifts elicits peripheral neuromuscular adaptations—such as stronger dorsiflexor recruitment and reduced extensor co-contraction—that translate into more symmetrical and energy-efficient walking (52). Collectively, these interconnected pathways provide a coherent physiological rationale for the functional improvements observed in the present study.

However, several limitations warrant acknowledgment. First, this study assessed only the short-term effects of a 6-week intervention, and the long-term retention of these improvements remains unclear. Future research should incorporate follow-up assessments to determine whether gait and balance improvements are sustained over time and whether continued VR training is necessary for maintaining functional gains. Second, the study was conducted in a controlled clinical environment, where patients trained on a treadmill with visually simulated walking challenges. While this provided a safe and structured setting, it may not fully replicate the real-world ambulatory challenges that stroke survivors face. Future studies should investigate the effectiveness of VR-based training in overground walking environments, incorporating diverse terrains, cognitive dual-tasking, and unpredictable obstacles to improve ecological validity. Third, all participants in this study were outpatients, making it challenging to standardize the conventional therapy administered to both groups. Although efforts were made to ensure consistency in the intervention protocols, variations in therapist approaches and individualized therapeutic plans may have influenced the results. Future studies should consider a more controlled rehabilitation setting or a crossover study design to minimize potential variability in conventional therapy across participants. Furthermore, the total intervention time differed between groups (60 min in the VTT arm vs. 30 min in the CT arm). While this disparity reflects the add-on nature of our efficacy design, the unequal dose prevents complete separation of training-content effects from time-dose effects. A future time-matched randomized trial is therefore warranted to verify that the observed benefits are attributable to the virtual reality-based treadmill gait training compared to other training.

## 5 Conclusion

This study demonstrated the positive efficacy of VR-based treadmill gait training in improving gait performance, functional mobility, and balance in chronic stroke patients. Notably, significant improvements were observed even in long-term chronic stroke survivors with persistent gait impairments, suggesting the effectiveness of VR-based rehabilitation beyond the early recovery phase. The ability to simulate diverse virtual walking environments in a controlled setting highlights its potential to enhance functional mobility and reduce falling risks in real environments. Thus, VR-based treadmill training may serve as a valuable rehabilitation approach for chronic stroke patients; however, further research is needed to explore its long-term benefits and optimal implementation strategies.

## Data Availability

The datasets presented in this article are not readily available due to participant confidentiality. Requests to access the datasets should be directed to hansh0106@yuhs.ac.
